# One-Day Antibiotic Infusion for the Prevention of Postoperative Infection Following Arthroplasty: A Case Control Study

**DOI:** 10.5402/2011/839641

**Published:** 2011-07-05

**Authors:** Rui Niimi, Masahiro Hasegawa, Goshin Kawamura, Akihiro Sudo

**Affiliations:** Department of Orthopaedic Surgery, Mie University Graduate School of Medicine, 2-174 Edobashi, Tsu City, Mie 514-8507, Japan

## Abstract

Intravenous antibiotics effectively reduce the prevalence of postoperative infection. However, Japanese orthopaedic surgeons have no consensus with regard to the optimal duration of prophylaxis. The aim of this study is to compare the outcome of one-day intravenous antibiotic administration with that of long-term intravenous antibiotic administration. Patients who underwent total hip or knee arthroplasty were divided into 2 groups to receive one of 2 prophylactic protocols retrospectively. Group A (223 patients) received intravenous antibiotics twice only on the day of surgery, whereas Group B (104 patients) received intravenous antibiotics for at least 3 days after surgery. We analyzed the wound infection rate and monitored liver and renal functions. None of these patients had a postoperative infection. No liver dysfunction and renal dysfunction were observed. One-day antibiotic infusion was as effective as long-term antibiotics in preventing infection after arthroplasty and achieved greater cost effectiveness.

## 1. Introduction

Infection is one of the most devastating complications associated with arthroplasty [1–7]. Prophylactic antibiotics have been proven to be an effective measure for the prevention of postoperative infection. There is no consensus with regard to the optimal duration of prophylaxis. The standard practice is to administer prophylactic intravenous antibiotics only on the day of surgery in Western countries [8–11]; however, in Japan, prophylactic intravenous antibiotics are administered for several days postoperatively [12], and one-day antibiotic infusion is rare. A questionnaire survey of Japanese orthopaedic surgeons showed that 86% of surgeons administered intravenous antibiotics for 7 days or longer after prosthetic surgery [12]. We have no precise guidelines on the prophylactic use of antibiotics in Japanese orthopaedic surgery.

At our institution, since July 2004, antibiotics have been infused only on the day of primary total hip arthroplasty (THA) and total knee arthroplasty (TKA). Here, we retrospectively compared the outcome of one-day intravenous antibiotic administration with that of long-term intravenous antibiotic administration.

## 2. Materials and Methods

223 patients who underwent arthroplasties received intravenous antibiotics only on the day of surgery (Group A). Another 104 patients who underwent arthroplasties received intravenous antibiotics for at least 3 days after surgery (Group B; control group). The characteristics of the groups are shown in Table 1. No significant differences were found in terms of age, body mass index (BMI), sex, procedures, and operative time between the groups. The mean follow-up periods were 32 ± 16 months in Group A and 51 ± 23 months in Group B. Patients who underwent revision arthroplasty and those who were susceptible to infection due to factors such as diabetes mellitus, rheumatoid arthritis, or steroid therapy, were excluded from this study. This study was reviewed and approved by the local ethics committee.

Surgery was performed in operating rooms equipped with vertical laminar airflow. The surgical team used a total body exhaust system. The skin of the surgical field was not shaved but was prepared with 10% povidone iodine solution twice before draping. All THAs were performed by one surgeon (A. Sudo), and all TKAs were performed by another one surgeon (M. Hasegawa). We performed THA with cementless prostheses; however, we used only cemented prostheses in TKA. No antibiotic-impregnated cement was used. Drains were routinely used and removed 2 days after surgery. Sutures were removed 14 days after surgery.

**Figure 1:**
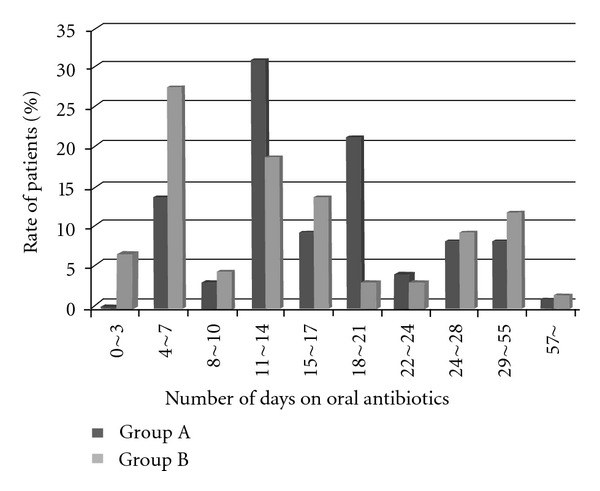
Period of time for oral antibiotic.

For both groups, antibiotics were intravenously infused starting 30 minutes before surgery, with the second administration approximately 6 hours later [13]. The doses of intravenous antibiotics were 2-3 grams per day according to the instructions. In Group A, antibiotics were intravenously infused on the day of surgery, and oral antibiotics were administered for 5 to 77 days postoperatively (average, 18 days). In Group B, antibiotics were intravenously infused for 3 to 24 days with an average of 8 days (twice daily in the morning and at night), and oral antibiotics were administered for 3 to 72 days postoperatively with an average of 16 days. Oral antibiotics were administered when the serum C-reactive protein (CRP) level dropped below 1.0 mg/dL (Figure 1). In Group A, PIPC (piperacillin, *β*-lactam antibiotics; Taisho-Toyama Pharmaceutical Co. Ltd, Tokyo, Japan) was used in 97 patients, CEZ (cefazolin, a first-generation cephalosporin; Otsuka Pharmaceutical Co. Ltd., Tokyo, Japan) was used in 72 patients, SBT/ABPC (sulbactam sodium/ampicillin sodium, a combination drug of ampicillin and *β*-lactamase inhibitor; Pfizer Japan Inc., Tokyo, Japan) was used in 30 patients, CTM (cefotiam, a second-generation cephalosporin; Takeda Pharmaceuticals Co. Ltd., Osaka, Japan) was used in 12 patients, FMOX (flomoxef, a second-generation cephalosporin; Shionogi & Co. Ltd.) in 9 patients, and others were in three patients. CFPN-PI (cefcapene pivoxil, an oral cephalosporin; Shionogi & Co. Ltd., Osaka, Japan) was then administered orally. In Group B, CEZ was used in 38 patients, PIPC in 28 patients, ABST/SBT in 10 patients, FMOX in 10 patients, CTM in 9 patients, and others in 9 patients were intravenously infused, followed by oral administration of CFPN-PI.

We evaluated the presence of wound infection from signs such as persistent fever, localized fever, localized redness, or purulent discharge and nonwound infection, such as respiratory or urinary tract infection. We monitored liver and renal functions as well as inflammatory markers including serum CRP and white blood cell count (WBC).

The study was conducted according to the ethical principles stated in the Declaration of Helsinki and local regulations. Written informed consent was obtained from all patients. 

### 2.1. Statistical Analysis

We compared the patients' baseline characteristics using the Mann-Whitney *U* test and chi-squared test. The analysis was performed using the StatView statistical software package (version 5.0; SAS Institute, Cary, NC, USA).

## 3. Results

None of these patients developed wound infection or nonwound infection during the followup for a minimum 12 months after surgery. We found liver function test abnormalities in 67 of the 223 patients (30%) in Group A and 26 of the 103 patients (25%) in Group B. There is no significant difference between group A and B (*P* > .05). In Group A, 8 patients had 2-fold and 4 patients had more than 3-fold elevation of aspartate aminotransferase (AST). 15 patients showed 2-fold and 11 patients had more than 3-fold elevation of alanine aminotransferase (ALT). In Group B, 4 patients had 2-fold and 3 patients had more than 3-fold elevation of AST. 11 patients had 2-fold and 6 patients had more than 3-fold elevation of ALT. No patients presented jaundice. No renal dysfunction was observed.

**Figure 2:**
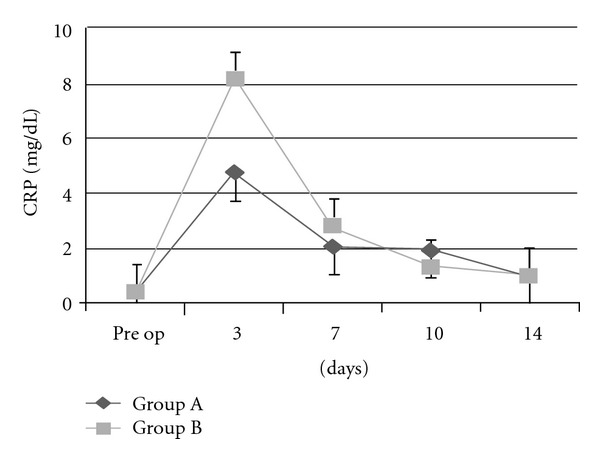
C-reactive protein (CRP) levels after arthroplasty. Values are expressed as mean ± standard deviation.

At 3 days after surgery, the mean CRP level was elevated to 9.7 mg/dL for Group A and 8.1 mg/dL for Group B, but at 7 days after surgery, it had dropped markedly to 2.9 mg/dL for Group A and 2.8 mg/dL for Group B and did not increase again (Figure 2).For both groups, WBC was elevated the day after surgery. However, it normalized at 3 days after surgery in most patients: 6445/mL for Group A and 6241/mL for Group B. None of the patients exhibited persistent fever.

## 4. Discussion

When first- and second-generation cephalosporins were infused only on the day of surgery, the incidence of deep infection ranged from 0 to 1.6%, and no significant difference was observed between one-day and long-term preventive antibiotic therapy [14–18]. A recent systemic review has shown that there is no advantage to prolonging the antibiotic prophylaxis for arthroplasty beyond 24 hours [19]. A study in the Norwegian arthroplasty register showed that the results were better if antibiotics were given 4 times on the day of surgery, as compared to once, twice, or 3 times. No further improvement was found in the patients given antibiotics infusion for 2 days or 3 days when compared to systemic prophylaxis 4 times in one day [20]. Others have advocated maintaining antibiotics therapy until drainage devices are removed from the wound [14]. A pronounced rise of the CRP level on the third postoperative day after arthroplasty is normal, but a further rise at one to 2 weeks suggests the presence of a serious complication, including infection [15]. If we had found an upward trend in the CRP level after the third postoperative day, we would have continued the long-term intravenous administration of antibiotics. None of the patients in the present study developed postoperative infection; hence, one-day infusion of antibiotics on the day of surgery appears to be effective in preventing infection.

Problems with long-term intravenous antibiotic administration include the emergence of drug-resistant bacteria, drug-induced hepatopathy and nephropathy, and high medical cost [16, 21, 22]. In the present study, Group B showed an increased incidence of liver function test abnormalities, thus suggesting an association between long-term antibiotics infusion and liver function test abnormalities. Regarding medical costs, the average daily cost of intravenous CEZ and CTM is 2,582 Japanese yen (about $28.7), while that of oral CFPN-PI is only 283 Japanese yen (about $3.1). Because the average length of infusion for Group B was 8 days, the difference in cost between Groups A and B was 16,083 Japanese yen (about $179). By intravenously administering antibiotics only on the day of surgery, it is possible to lower medical costs, and this is beneficial in terms of hospital management from the viewpoint of comprehensive medicine.

The limitations of this study include its small sample size and the effect of the oral administration of antibiotics. The numbers of patients were small to evaluate a difference in outcomes. In addition, many kinds of intravenous antibiotics were used, so the number of patients in each antibiotics group was small to perform detail statistical analyses. Because drug-resistant bacteria increase in association with the quantity of antibiotics, and contact with antibiotics in low concentration confers a risk of acquiring resistance, the administration of oral antibiotics might not be needed as prophylaxis against surgical site infection [23].

## 5. Conclusion

One-day antibiotic infusion was as effective as long-term antibiotic infusion in preventing infection after arthroplasty. Long-term intravenous infusion of antibiotics was associated with a higher incidence of liver function test abnormalities. In addition, one-day intravenous infusion of antibiotics on the day of surgery achieved greater cost effectiveness.

## Figures and Tables

**Table 1:** Characteristics of the patients.

		Group A	Group B	*P* value
Age	(years)	65.0 ± 10.8	63.7 ± 13.1	.826^a^
BMI	(Kg/m^2^)	24.6 ± 3.8	24.4 ± 4.0	>.999^a^
Gender	Female	190	80	.0661^b^
	Male	33	24	
Procedures	THA	153	63	.1531^b^
	TKA	70	41	
Operative time	(min)	108.4 ± 37.0	114.8 ± 42.2	.240^a^

Values are mean ± standard deviation. BMI, body mass index;

THA, total hip arthroplasty; TKA, total knee arthroplasty

^a^Mann-Whitney *U* test; ^b^Chi-squared test.
